# High dose methylphenidate treatment in adult attention deficit hyperactivity disorder: a case report

**DOI:** 10.1186/1752-1947-6-125

**Published:** 2012-05-14

**Authors:** Michael Liebrenz, Danielle Hof, Anna Buadze, Rudolf Stohler, Dominique Eich

**Affiliations:** 1 Psychiatric University Hospital, Research Group on Substance Use Disorders, Selnaustrasse 9, 8001, Zurich, Switzerland; 2 Psychiatric University Hospital, Department of Forensic Psychiatry, Lenggstrasse 31, 8032, Zurich, Switzerland; 3 University Hospital Zurich, Institute for Clinical Chemistry, Raemistrasse 100, 8091, Zurich, Switzerland; 4 Psychiatric University Hospital, Outpatient clinic for patients with ADHD, Lenngstr 31, 8032, Zurich, Switzerland

## Abstract

**Introduction:**

Stimulant medication improves hyperactivity, inattention, and impulsivity in both pediatric and adult populations with Attention Deficit Hyperactivity Disorder (ADHD). However, data regarding the optimal dosage in adults is still limited.

**Case presentation:**

We report the case of a 38-year-old Caucasian patient who was diagnosed with Attention Deficit Hyperactivity Disorder when he was nine years old. He then received up to 10 mg methylphenidate (Ritalin®) and 20 mg sustained-release methylphenidate (Ritalin SR®) daily. When he was 13, his medication was changed to desipramine (Norpramin®), and both Ritalin® and Ritalin SR® were discontinued; and at age 18, when he developed obsessive-compulsive symptoms, his medication was changed to clomipramine (Anafranil®) 75 mg daily. Still suffering from inattention and hyperactivity, the patient began college when he was 19, but did not receive stimulant medication until three years later, when Ritalin® 60 mg daily was re-established. During the 14 months that followed, he began to use Ritalin® excessively, both orally and rectally, in dosages from 4800-6000 mg daily. Four years ago, he was referred to our outpatient service, where his Attention Deficit Hyperactivity Disorder was re-evaluated. At that point, the patient’s daily Ritalin® dosage was reduced to 200 mg daily orally, but he still experienced pronounced symptoms of, Attention Deficit Hyperactivity Disorder so this dosage was raised again. The patient’s plasma levels consistently remained between 60–187 nmol/l—within the recommended range—and signs of his obsessive-compulsive symptoms diminished with fluoxetine 40 mg daily. Finally, on a dosage of 378 mg extended-release methylphenidate (Concerta®), his symptoms of Attention Deficit Hyperactivity Disorder have improved dramatically and no further use of methylphenidate has been recorded during the 24 months preceding this report.

**Conclusions:**

Symptoms of Attention Deficit Hyperactivity Disorder (ADHD) in this adult patient, who also manifested a co-occurring obsessive compulsive disorder, dramatically improved only after application of a higher-than-normal dose of methylphenidate. We therefore suggest that clinicians consider these findings in relation to their adherence to current therapeutic guidelines.

## Introduction

Attention Deficit Hyperactivity Disorder (ADHD) is a prevalent mental disorder characterized by symptoms of inattention (distractibility), hyperactivity, and impulsivity—all of which contribute to significant psychosocial impairment in the affected individuals [1]. Previously believed to be solely a disorder of childhood and adolescence, it is now accepted that about two-thirds of children diagnosed with ADHD will experience its symptoms in adulthood [2,3]. For example, adults suffering from ADHD are less frequently employed on a fulltime basis, have difficulty maintaining personal relationships, and are generally less satisfied with their families and their social and professional lives [4].

Prevalence of adult ADHD in the United States is estimated at around 4.4%, and the disorder is highly co-morbid with many other DSM IV disorders [1,5,6]. Although clinicians widely agree that a treatment approach which combines psychotherapeutic and pharmacological interventions is optimal, the prescription of stimulant medication is clearly the first line of treatment and is known to improve symptoms in both pediatric and adult populations [7]. In particular, the use of methylphenidate (MPH), has proved an efficacious pharmacological treatment for adult ADHD when administered in weight-adjusted doses equivalent to those used in a pediatric population [8].

Although the mechanism that underlies the action of stimulants in adult ADHD is still being investigated, the efficacy of both amphetamines and MPH is attributed to their ability to increase striatal and cortical dopamine levels [9]. While both drugs share common mechanisms of action, amphetamines increase dopamine release, whereas MPH inhibits re-uptake of dopamine by blocking the dopamine transporter (DAT) [9,10].

Oral formulations of MPH are available in short- and intermediate-acting preparations, as well as slow-release and long-acting ones. Oral administration of immediate-release MPH reaches peak plasma concentration after two hours, and decreases thereafter [5].

As a consequence, immediate-release MPH must be taken several times a day—and, even so, spikes in plasma level occur. On the other hand, long-acting formulations, such as once-daily osmotic release oral system (OROS) MPH (Concerta®), improve patient compliance and demonstrate efficacy similar to that of an immediate-release dose of MPH three times daily [11]. Data on the optimal dosage of these stimulants in adults is still limited, with upper-limit recommendations usually being 1 mg/kg for methylphenidate and 0.5 mg/kg for dexamphetamine [8].

## Case presentation

We report the case of a 38-year-old Caucasian man, with dual citizenship in the USA and Switzerland, who has been in treatment at the outpatient service of the Psychiatric University Hospital for the last four years. During childhood and adolescence, his family frequently relocated from Switzerland to the USA, and vice versa. His father, an entrepreneur, described the patient as being an “uneasy” child who was different from his siblings, and he had sought professional help for his son from a child psychiatrist in the USA. This psychiatrist diagnosed the patient, who was then nine years old, with ADHD, and over the course of the next five years, the patient received up to 10 mg of immediate-release MPH (Ritalin®). Later on, his dosage was increased to 20 mg of sustained-release MPH (Ritalin SR®) daily, which initially improved the patient’s symptoms of hyperactivity and inattention.

Twenty-five years ago, while he was still living in the USA, the patient’s medication was changed—for the same indication—to desipramine (Norpramin®), and MPH (Ritalin®) was discontinued. Nevertheless, most of his difficulties persisted throughout elementary- and middle school. Between ages 14 and 16, the patient was therefore sent to a school that specialized in the instruction of students with learning disabilities such as dyslexia. At age 18, he developed obsessive-compulsive traits and received counselling; his medication was also changed at that time to clomipramine (Anafranil®) 75 mg/daily.

Soon afterward, the family again relocated from the USA to Switzerland, where the patient attended high school—followed, in 1993, by college in the USA. Still suffering from symptoms of inattention and hyperactivity, he did not finish his studies at this college, and failed in several other attempts to obtain a secondary degree in the USA, Great Britain, and Switzerland. The patient recalled that he never seemed able to “focus.”

For unknown reasons, he did not receive further stimulant medication until six years ago, when a Swiss psychiatrist prescribed immediate-release MPH (Ritalin®) 60 mg/daily to reduce the patient’s distractibility at his newfound job as a sales clerk. The patient experienced MPH as highly effective, but not sustainable throughout the course of a day. During the following 14 months, he therefore began to use Ritalin® excessively, both orally and rectally, in dosages up to 4800-6000 mg/daily, by applying to thirty different doctors in three different cantons of Switzerland. He thereby fulfilled the criteria for stimulant dependence, although he never used any substance other than MPH.

Four years ago, the patient was referred to our outpatient service for treatment. He received clinical diagnostic interviewing (SCID I, SCID II) and his ADHD was re-evaluated using recommended practice parameters [12]. In addition, his plasma levels of methylphenidate were obtained, a routine medical workup was performed, and information on his past medical history was collected.

Physical examination revealed chronic back pain, and a local rheumatologist diagnosed ankylosing spondylitis (Bekhterev's disease). No other pathology was found, nor did the patient have any history of palpitations, tachycardia and dyspnea, or other adverse cardiovascular effects commonly associated with stimulant use. His blood pressure and heart rate were within the normal range, and an ECG showed no abnormalities.

The diagnostic (SCID) testing confirmed the presence of an obsessive-compulsive disorder and revealed a combined personality disorder. There was no further history of substance misuse or dependency; the patient reported only a recreational use of cannabis. The patient first received immediate-release MPH 200 mg. This was later changed to 240 mg, and finally to 270 mg MPH per day. We repeatedly checked the plasma levels of MPH in the blood under all these dosages and found them to be within the reference range. Moreover, when the patient received extended-release MPH at a dosage of 378 mg/day, his blood plasma remained within reference range. The reference range is 43-257 nmol, and we found his plasma levels to be between 60 nmol-187 nmol/l.

Using a combined psychosocial (CBT) and pharmacotherapeutic treatment approach, the patient’s daily Ritalin® dosage could initially be reduced to 200 mg/daily orally. However, the patient still experienced pronounced symptoms of ADHD at this dosage, and these symptoms were also reported by his family members. He exhibited high levels of distractibility both within the clinical setting (e.g., during group therapy), and at home, where he was unable to help around the house or contribute to the care of his newborn child.

While his signs of OCD diminished with fluoxetine 40 mg/daily, his ADHD symptoms only improved dramatically after his dosage was increased to 378 mg of extended-release MPH (Concerta®). This was reflected in an increase of his score on the Global Assessment of Functioning scale from 43 to 68; and at home, the patient was able to care for his child several days a week and re-establish interpersonal relationships. No further excessive use of methylphenidate has been recorded for the past 24 months.

## Discussion

According to the NHS National Institute for Health and Clinical Excellence (NICE) 2008 guidelines for treatment of adult ADHD, MPH dosage should be increased gradually until further improvement of ADHD symptoms ceases (e.g., symptom reduction, behavior change, improvements in education and/or relationships) [13]. These same guidelines set the maximum dose at 100 mg MPH/day.

Since we had seen improvement in our patient only at a higher-than-recommended dosage, we not only monitored for side effects (e.g., manic symptoms, psychosis, and suicidal ideation), all of which were non-existent—we also obtained the patient’s plasma levels of methylphenidate. After the addition of deuterated methylphenidate as an internal standard, methylphenidate was obtained from serum samples by liquid-liquid extraction, and was subsequently measured by chromatographic separation on C18 reversed-phase HPLC columns (Interchim, France) and electrospray ionisation mass spectrometry [14].

Using Positron Emission Tomography (PET) examination, Swanson et al. demonstrated a high correlation between peak plasma concentration of the d-isomer of methylphenidate (d-MPH) and dopamine transporter occupancy, and concluded that plasma concentration of d-MPH served as a good measure of brain levels of methylphenidate [15]. The same authors estimated that the dose required to block 50% of the DAT in adults was 0.25 mg/kg.

Since our patient had been prescribed the racemic mixture of MPH (1:1 ratio for d:I enantiomers), we were only able to obtain and evaluate d,I-MPH plasma levels – all of which were within reference range: 43 – 257 nmol/l (d,I-MPH). To compare our results with the findings of Swanson et al., we therefore calculated d-MPH plasma levels, which indicated that our patient’s brain levels of methylphenidate were probably also within the norm.

Unlike many other substances, which are metabolized by hepatic cytochrome P450 (CYP) enzymes, MPH is converted to inactive ritalinic acid by carboxylesterase (CES). Liver carboxylesterase 1 (CES1) is responsible for the hydrolysis of both d- and l-isomers of MPH,including the first-pass metabolism of this substance [16]. Although there is evidence that specific CES1 gene variants may exist, which could lead to clinically significant alterations in pharmacokinetics and drug response of carboxylesterase 1 substrates, only two CES1 variants have been identified to our knowledge, both of which result in reduced enzyme activity [17,18]. Furthermore, the presence of one or two of these variants would not explain our findings, since their presence would result in higher plasma levels of MPH, not lower ones. While we cannot rule out the possibility this patient possesses a CES1 gene variant with an increased enzyme activity, no genetic testing is currently available to confirm or disconfirm this assumption.

An illusory positive bias has been reported on the part of both children and adults with ADHD, suggesting that patients with ADHD are not the most reliable source of their own psychosocial functioning [3]. We therefore relied primarily on physician ratings and family reports to determine our patient’s response to MPH, and relied only secondarily on his self-rating.

It is now widely accepted that the use of methylphenidate within recommended dose-ranges offers a safe form of treatment despite possible increases in patients’ heart rate and blood pressure. In fact, a recent retrospective, population-based cohort study by Habel et al., on a total of 150,359 young and middle-aged adults with a current or new use of ADHD medication, did not find an increased risk of serious cardiovascular events [19]. However, since cases of sudden cardiac death have been reported when prescribing methylphenidate in combination with other drugs (e.g., clonidine) [20], we closely monitored for adverse cardiovascular effects, paying special attention to potential increases in blood pressure and heart rate. Despite the high dose of MPH that this patient received, we found no significant changes in these parameters.

Results of an earlier study in mice suggest that beta 2-adrenergic agonist drugs are capable of potentiating methylphenidate-induced hepatotoxicity by increasing hepatic methylphenidate concentrations [21]. However, we found only one case report, from 1972, in which hepatotoxicity was associated with use of methylphenidate [22], and we found no elevated liver enzymes in our patient during his treatment.

Because of its potential for misuse, some clinicians discourage use of methylphenidate in ADHD patients with a co-occurring substance use disorder (SUD). However, analyses from two randomized placebo-controlled trials among adolescents with a SUD concluded that extended-release MPH can be safely administered, despite non-abstinence, when it is closely monitored [23].

Data were not available to us on the use of high doses of MPH especially among the elderly or those patients with co-morbid heart or liver conditions. But data that stem primarily from poison centers show that the most common symptoms in patients with methylphenidate abuse were tachycardia, agitation/irritability, and hypertension. Outcomes were reported to be good, especially in cases only involving methylphenidate abuse [24].

Moreover, a recent Swiss study of 14 patients with self-reported MPH abuse of 30-400 mg MPH found that oral or nasal MPH abuse was associated with only minor to moderate sympathomimetic toxicity, which was generally self-limited and sometimes treated with sedatives [25]. And the case study of a 14-year-old girl who had ingested 21 long-acting methylphenidate 54-mg tablets (1,134 mg Concerta®) described a sympathomimetic syndrome with agitation, visual hallucinations, slight hypertension, and sinus tachycardia, but no life-threatening symptoms [26]. We did not observe any such symptoms in our patient.

## Conclusions

In this adult patient, who has ADHD, an obsessive-compulsive disorder, and a combined personality disorder, we observed an excessive use of methylphenidate, which fulfilled the criteria of dependence at the time of his admission to our outpatient services. Clinically, we observed dramatic improvement of his ADHD symptoms only after application of a higher-than-normal dose of MPH.

To our knowledge, this is the first reported case of high-dose treatment in a patient with adult ADHD. We therefore suggest that clinicians consider these findings in their work with patients when ADHD symptoms do not improve sufficiently with currently recommended dosages of stimulants. Because no long-term experiences with high-dose methylphenidate treatment have been published to date, we further advise clinicians to monitor clinical symptoms when using high doses of MPH, despite the fact that we did not find a sympathomimetic syndrome.

## Patient’s perspective

“As a child, I was always getting into trouble for 'having too much energy.' I never understood why people were always moving slowly. My parents and teachers were so frustrated with me that I couldn't organize myself or learn things at the same pace as 'everyone else.’ It was decided that I would try Ritalin, but it never lasted long enough to make that much of an improvement and I stopped taking it.

As an adult, the demands from my relationships became more intense, as did everyone's frustrations, including my own. The pressure to try and solve my focus problems and impulsive behaviors became so great that my family encouraged me to try an ADHD medicine again. I was prescribed a 'normal' dose of Concerta, and it felt like a light bulb had been turned on in my head. Suddenly I was able to concentrate long enough to read, began keeping appointments and coping better with day to day activities. Caring for our son became more manageable as I was able to plan and organize his day without becoming totally overwhelmed.

But soon after I started the medicine, I noticed the positive focus wore off so quickly between doses that I felt I required a higher dose in order to maintain focus for the entire day. It was difficult for me to explain this need to the medical community as it was something that was out of the ordinary apparently. All I knew was that it just 'wore off too quickly.’ Under close observation the dosage was increased, eventually to 7 tablets a day. Through blood work it was proven that my body was processing the medicine at a normal level.

Although I still experience focus issues from ADHD, the higher dosage of the medicine has been an aid in helping me manage and cope with those disabilities.”

## Consent

Written, informed consent was obtained from the patient for publication of this case report and accompanying graphs. A copy of this consent is available for review by the Editor-in-Chief of this journal.

## Abbreviations

ADHD: Attention deficit hyperactivity disorder; CBT: Cognitive behavioral therapy; DSM-IV: Diagnostic and statistical manual of mental disorders; DAT: Dopamine transporters; ECG: Electrocardiography; GAF score: Global assessment of functioning score; MPH: Methylphenidate; NICE: NHS national institute for health and clinical excellence; OCD: Obsessive compulsive disorder; SCID: Structured clinical interview for DSM-IV.

## Competing interests

The authors declare that they have no competing interests*.*

## Authors’ contributions

AB, ML, and RS were involved in the treatment process of this patient, performed the medical workup, contributed to clinical psychological testing, and summarized the medical history. DH evaluated the lab results. ML and DE were the major contributors in writing the manuscript. All authors evaluated the findings, and read and approved the final manuscript.
